# Characterization of the Doublesex/MAB-3 transcription factor DMD-9 in *Caenorhabditis elegans*

**DOI:** 10.1093/g3journal/jkac305

**Published:** 2022-12-01

**Authors:** Rasoul Godini, Roger Pocock

**Affiliations:** Development and Stem Cells Program, Monash Biomedicine Discovery Institute and Department of Anatomy and Developmental Biology, Monash University, Melbourne, Victoria 3800, Australia; Development and Stem Cells Program, Monash Biomedicine Discovery Institute and Department of Anatomy and Developmental Biology, Monash University, Melbourne, Victoria 3800, Australia

**Keywords:** *Caenorhabditis elegans*, transcription factor, expression analysis, behavioral analysis, neurobiology

## Abstract

DMD-9 is a *Caenorhabditis elegans* Doublesex/MAB-3 Domain transcription factor (TF) of unknown function. Single-cell transcriptomics has revealed that *dmd-9* is highly expressed in specific head sensory neurons, with lower levels detected in non-neuronal tissues (uterine cells and sperm). Here, we characterized endogenous *dmd-9* expression and function in hermaphrodites and males to identify potential sexually dimorphic roles. In addition, we dissected the *trans-* and *cis*-regulatory mechanisms that control DMD-9 expression in neurons. Our results show that of the 22 neuronal cell fate reporters we assessed in DMD-9-expressing neurons, only the neuropeptide-encoding *flp-19* gene is cell-autonomously regulated by DMD-9. Further, we did not identify defects in behaviors mediated by DMD-9 expressing neurons in *dmd-9* mutants. We found that *dmd-9* expression in neurons is regulated by 4 neuronal fate regulatory TFs: ETS-5, EGL-13, CHE-1, and TTX-1. In conclusion, our study characterized the DMD-9 expression pattern and regulatory logic for its control. The lack of detectable phenotypes in *dmd-9* mutant animals suggests that other proteins compensate for its loss.

## Introduction

*Caenorhabditis elegans* is a microscopic nematode that generates 2 sexes: hermaphrodites and males. Despite core similarities, each sex possesses sexually dimorphic structures, nervous systems, and behavior (Shen and Hodgkin 1988; Yi *et al.* 2000; Lints and Emmons 2002; Lee and Portman 2007; Oren-Suissa *et al.* 2016; Serrano-Saiz *et al.* 2017; Cook *et al.* 2019; Bayer *et al.* 2020). Hermaphrodites and males have 302 and 387 neurons, respectively, of which 294 neurons are common between sexes (Sulston *et al.* 1980; Hobert 2005; Barr *et al.* 2018). Sex-specific neurons such as HSN neurons in hermaphrodites and the ray neurons in males control sex-related behaviors, such as egg-laying and mating, respectively (Trent *et al.* 1983; Shen and Hodgkin 1988; Liu and Sternberg 1995; Lints and Emmons 2002). Moreover, neuronal connectivity between some neurons is sex-specific and regulated following maturation (Oren-Suissa *et al.* 2016; Cook *et al.* 2019). In *C. elegans*, sex-specific characteristics are regulated by the sex determination pathway and members of the Doublesex/MAB-3 Domain (DMD)/DMRT family of transcription factors (TFs; Shen and Hodgkin 1988; Yi *et al.* 2000; Wolff and Zarkower 2008; Oren-Suissa *et al.* 2016).

There are 11 DMD TFs encoded in *C. elegans* that regulate various sexually dimorphic characteristics (Matson and Zarkower 2012). DMD TFs are expressed in both sexes but may exhibit sex-specific expression patterns. For example, MAB-3 is expressed in the male ADF chemosensory neurons but not in hermaphrodites (Yi *et al.* 2000; Fagan *et al.* 2018). Likewise, DMD-3 is only expressed in the male PHC sensory neuron (Serrano-Saiz *et al.* 2017), and the DMD-5/DMD-11 TFs are only expressed in the male AVG interneuron (Oren-Suissa *et al.* 2016). Conversely, DMD-4 is expressed in the PHA and PHB neurons of adult hermaphrodites but not adult males (Bayer *et al.* 2020). These sexually dimorphic expression patterns regulate sex-specific characteristics, such as the expression of gene batteries, axo-dendritic structures, and synaptic connectivity, all of which modulate animal behavior.

DMD TFs can regulate sexual dimorphism across an organism or in specific cellular contexts. MAB-3 is required for male V ray development, yolk development in males, male-specific cell lineages, and male behavior (Shen and Hodgkin 1988; Yi *et al.* 2000). MAB-23 is also required for the development of male sexual characteristics, including ray dopaminergic neuron patterning, axon pathfinding, and mating behavior (Lints and Emmons 2002). DMD-3 controls male-specific PHC neuron characteristics, such as development, synaptogenesis, and gene expression (Mason *et al.* 2008; Serrano-Saiz *et al.* 2017). DMDs also regulate sexual dimorphic connectivity in which synapses between sex-shared neurons are sex-specific. For example, the PHB and AVG neurons maintain synaptic connections in adult males but not adult hermaphrodites (Oren-Suissa *et al.* 2016; Cook *et al.* 2019). In males, DMD-3 is required for PHC axon extension and male-specific expression of the FMRF-amide neuropeptide gene *flp-11* (Serrano-Saiz *et al*. 2017). DMD-4 promotes synaptic pruning in male-specific synapses between the PHB and AVG neurons in hermaphrodites (Bayer *et al.* 2020). DMD-5 and DMD-11 control synaptic patterning of the PHB and AVG neurons in males by suppressing the pruning of male-specific synapses (Oren-Suissa *et al.* 2016). Moreover, DMD-5 and DMD-11 regulate AVG-mediated male mating behavior (Oren-Suissa *et al.* 2016), and DMD-10 regulates ASH-mediated responses to nose touch and high osmolarity (Durbeck *et al.* 2021).

Here, we focus on an uncharacterized member of the DMD TF family: DMD-9. Single-cell transcriptome analysis revealed that *dmd-9* is expressed in a subset of head sensory neurons (Taylor *et al.* 2021). DMD-9 is highly expressed in the BAG neurons, which sense oxygen (O_2_) and carbon dioxide (CO_2_) gases, and regulate exploration and egg-laying behavior (Ringstad and Horvitz 2008; Zimmer *et al.* 2009; Juozaityte *et al.* 2017). DMD-9 is also expressed in the AFD thermosensory neurons (Cassata *et al.* 2000), and the AWB/ASE/AWC chemosensory neurons (Bargmann and Horvitz 1991; Bargmann *et al.* 1993; Troemel *et al.* 1997). Here, we studied DMD-9 expression in both sexes through endogenous tagging with GFP. We also investigated the potential functions of DMD-9 in neuronal fate determination and function in hermaphrodites and males. We also studied the *trans-* and *cis-*regulatory mechanisms that regulate *dmd-9* expression in the head neurons. We show that DMD-9 is not expressed in a sexually dimorphic manner in head sensory neurons (BAG, AFD, AWB, AWC, and ASE). However, it is expressed in non-neuronal sex-specific tissues: uterine cells in hermaphrodites and sperm in males. We found that the expression of neuron cell fate reporters in DMD-9-expressing neurons is not dependent on DMD-9, with the exception of the FMRF-amide neuropeptide *flp-19* in the BAG neurons. Finally, we identified *cis-*regulatory elements and fate-determining TFs (ETS-5, EGL-13, CHE-1, and TTX-1) that drive *dmd-9* expression in head neurons. Taken together, our study characterized the expression pattern and regulation of DMD-9 in both *C. elegans* sexes. The lack of detectable phenotype upon DMD-9 loss suggests compensation by other proteins.

## Materials and Methods

### Strains and maintenance of the *C. elegans* strains

All strains were maintained on nematode growth medium (NGM) plates at 20°C as described previously (Brenner 1974). Strains used in this study are listed in Supplementary Table 1. Animals were synchronized to specific ages by picking.

### Molecular analysis

#### CRISPR-Cas9

A coding sequence GFP-AID-TEV-FLAG was knocked in to the last exon of *dmd-9* gene using CRISPR-Cas-9 (Dokshin *et al.* 2018). We designed the crRNA to guide the CRISPR-Cas-9 enzyme and a donor DNA fragment containing the insertion and overhangs to be inserted in the location of interest (https://sg.idtdna.com/sgRNA; Supplementary Table 2). CRISPR-Cas-9 mixture was injected into wild-type N2 animals and the F1 progeny were screened by genotyping and microscopy for GFP expression.

#### Cloning

Cloning and mutagenesis were performed using In-Fusion restriction-free cloning (Takara). Reporter gene constructs were cloned by inserting PCR-amplified promoter elements into the pPD95.75 vector (Fire Vector Kit). Constructs were confirmed by Sanger sequencing.

#### Transgenic lines

Transgenic lines were generated by injecting constructs into young adult hermaphrodite N2 animals. *Pmyo-2::RFP*; *Punc-122::RFP* or *Punc-122::GFP* were used as co-injection markers. For reporter constructs, 30–50 ng/µl of the construct was injected. For rescue lines, 1–5 ng/µl of *ets-5* and *egl-13* fosmids was injected into *ets-5(tm866); him-8(e1489)* and *egl-13(ku194); him-8(e1489)*, respectively.

#### Auxin-inducible degron analysis

Experiments were performed as described (Zhang *et al.* 2015). For the depletion of auxin-inducible degron (AID) tagged proteins, we used 0.1 or 1 mM auxin. For recovery experiments, we exposed the animals to 0.1 mM auxin for 24 h followed by 24 h incubation on standard NGM plates. In all experiments, NGM plates were seeded with *Escherichia coli* OP50 bacterial lawns.

### Microscopy

Animals were mounted in sodium azide (NaN_3_) 25 µM. A Zeiss AXIO Imager M2 fitted with an Axiocam 506 mono camera and Zen 2 pro software (Zeiss) was used to take fluorescent and DIC images. Images were analyzed using Zen 2 pro software (Zeiss) and ImageJ v1.53c. Corrected Total Cell Fluorescence is calculated as integrated density–(area × mean gray of background).

### Behavioral experiments

#### Chemotaxis

Chemotaxis behavior was performed on 90 mm agar plates according to (Bargmann *et al.* 1993) with some modifications. For each replicate, 50–100 animals were used. In the case of animals carrying *him-8(e1489)* or *him-5(e1490)* mutations, 200–300 L4 males were transferred to a new plate to grow into an adult. Depending on the chemical, different concentrations from 1:10 to 1:10,000 (V/V) were freshly diluted in absolute ethanol (Merck). For NaCl chemotaxis, 1.0 and 0.25 M concentrations were used (Bargmann and Horvitz 1991). To calculate the chemotaxis index, the following formula was used:

$$\text{Chemotaxis index}\;=\;\frac{\text{(no}\text{. of animals on the chemical-no}\text{. of animals on the control)}}{\text{total no}\text{.of animalson a plate}}$$

#### Gas sensing

Gas sensing was performed as previously described (Romanos *et al.* 2015). Animals were exposed to low concentrations of O_2_ (10% compared with 21% in control) or high concentrations of CO_2_ (1% compared with 0% in control) for 6 min. The proportion of gases was balanced by changing the N_2_ level. Animal motility was recorded with a 4-megapixel CCD camera (Jai) and analyzed using MatLab-based image processing to obtain instantaneous speed during continuous forward movements (Ramot *et al.* 2008; Tsunozaki *et al.* 2008).

#### Exploration

Exploration behavior was assayed as previously described (Flavell *et al.* 2013) with some modifications. Assay plates were 60 mm NGM plates uniformly seeded with *E. coli* OP50. Strains were grown well-fed for 2 generations at 20°C prior to each experiment. Exploration was assayed for young adult hermaphrodites and males for 16 and 2–3 h, respectively. Animals were scored based on the count of squares entered in an 86-grid area.

#### Food leaving

Food-leaving behavior was performed as previously described (Lipton *et al.* 2004). Experiments were performed on 90 mm plates spotted with 18 µl of *E. coli* OP50 (OD_600_ = 1.0) culture grown for 12–16 h at room temperature. To synchronize animals, 20–30 L4 hermaphrodite or male animals of each strain were sex-segregated and matured for 12 h. Individual animals were placed on the *E. coli* OP50 spot and scored for leaving events at 4, 8, 12, and 24 h. A leaving event was scored if the animal reached 3 cm from the edge of the seeded bacteria (Lipton *et al.* 2004). To analyze the results, a survival model was applied to compare the ratio of leaver animals between different strains. The statistical significance of the experiment was calculated with the Gehan–Breslow–Wilcoxon test.

#### Egg-laying

Egg-laying behavior was performed as previously described (Ringstad and Horvitz 2008). Five adult hermaphrodites, synchronized from L4 for 30 h, were transferred to assay plates containing 40 µl of freshly-coated *E. coli* OP50 culture seeded at the center of NGM plates and left to lay eggs for 1 h at 20°C. The developmental stages of eggs laid were divided into Stages 1–6 (Ringstad and Horvitz 2008) and analyzed using a high-power dissecting microscope (Olympus SZX16). The distribution of egg developmental stages was analyzed using Wilcoxon Mann–Whitney rank-sum test.

### Bioinformatic analysis

Transcriptomic analysis of *dmd-9* gene expression was obtained from (cengen.shinyapps.io/CengenApp/; Taylor *et al.* 2021) and visualized using R programming. The JASPAR program (jaspar.genereg.net/) was used to predict TFs binding elements within the *dmd-9* promoter (Castro-Mondragon *et al.* 2022).

### Statistical analysis

Statistical analysis and visualization of plots were performed using GraphPad Prism software version 8.0.2 (GraphPad Software Inc.). A *P*-value <0.05 was considered significant.

## Results

### DMD-9 is expressed in specific head sensory neurons in *C. elegans*

**Fig. 1.**
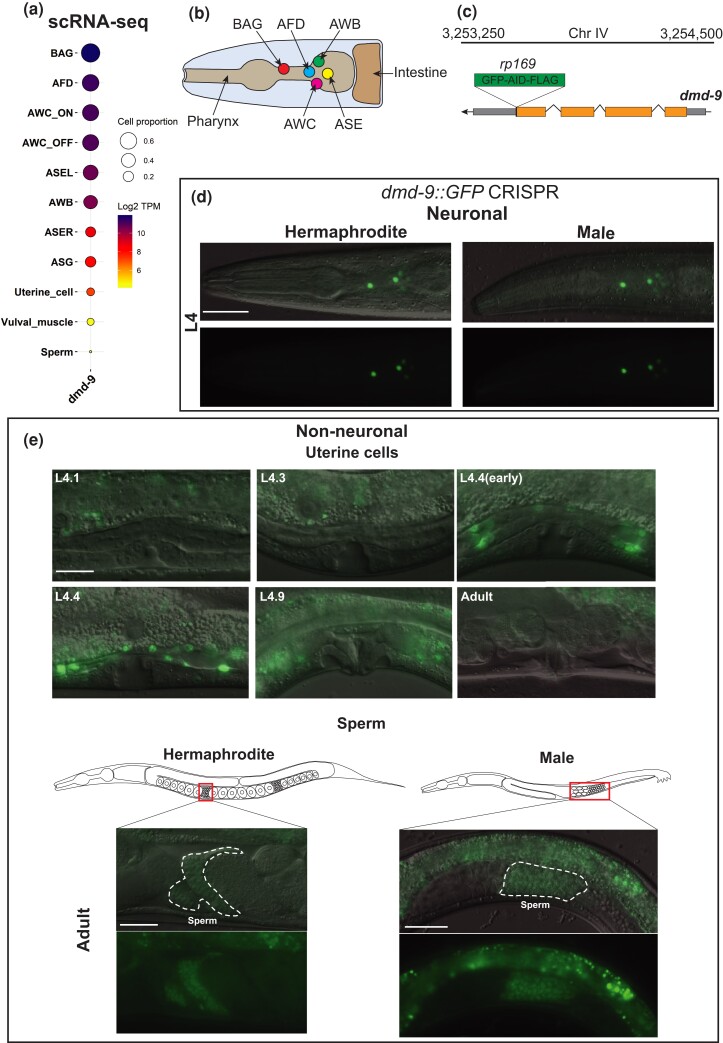
DMD-9 expression in hermaphrodites and males. a) Gene expression pattern of *dmd-9* in *C. elegans* obtained from (cengen.shinyapps.io/CengenApp/; Taylor *et al.* 2021). TPM = transcript per million. b) Schematic of head neurons that express *dmd-9*. View = left side (only 1 of each bilateral pair of neurons is shown). c) GFP-AID-FLAG coding sequence was endogenously inserted after the last exon of *dmd-9* gene (*rp169*) using CRISPR-Cas9 technology. d) Expression of *dmd-9::GFP(rp169)* in the head of hermaphrodites and males at L4 stage. Scale bar = 30 μm. Anterior to the left. e) Expression of *dmd-9::GFP(rp169)* in non-neuronal uterine cells and sperm. Uterine cells exhibit transient GFP expression through the L4 stage with the highest level at L4.4 sub-stage. Scale bar = 10 µm.

Single-cell transcriptome data from L4 hermaphrodites show that *dmd-9*, which encodes a member of the DMD family of TFs, is expressed in neuronal and non-neuronal cells. High *dmd-9* expression was detected in the BAG, AFD, AWC^on^, AWC^off^, ASEL, and AWB neurons, with lower expression in ASER, and the ASGs (Fig. 1a, b; Taylor *et al.* 2021). Additionally, *dmd-9* expression was detected in non-neuronal tissues, including uterine cells, vulva muscle, and sperm (Fig. 1a). Some members of the *C. elegans* DMD TF family (*dmd-3*, *dmd-5,* and *dmd-11*) show sexually dimorphic expression patterns in *C. elegans* (Oren-Suissa *et al.* 2016; Serrano-Saiz *et al.* 2017). To investigate the potential sex-specific expression pattern of DMD-9, we used CRISPR-Cas9 to endogenously tag the DMD-9 C-terminus with GFP and studied expression throughout development in both hermaphrodites and males (Fig. 1c–e). We detected DMD-9::GFP expression in 10 head neurons of hermaphrodites at the L4 stage (Fig. 1d). We also observed weak and inconsistent expression in other head neurons and transient weak expression in the tail of hermaphrodites and males at the L4 stage (not analyzed). No overt differences were observed in DMD-9::GFP expression in neurons of hermaphrodites and males (Fig. 1d). We also detected DMD-9::GFP expression in non-neuronal tissues. Transient DMD-9::GFP expression was observed in the uterine cells at specific sub-stages of the L4 stage. DMD-9 is not expressed from L4.0 to L4.3, with expression first detected in the early L4.4 stage, with the highest level during L4.4 before gradual loss by the L4.9 stage (Mok *et al.* 2015; Fig. 1e). DMD-9::GFP was not detected in adult uterine cells (Fig. 1e). We also detected DMD-9::GFP expression in sperm of hermaphrodites and males (Fig. 1e).

**Fig. 2.**
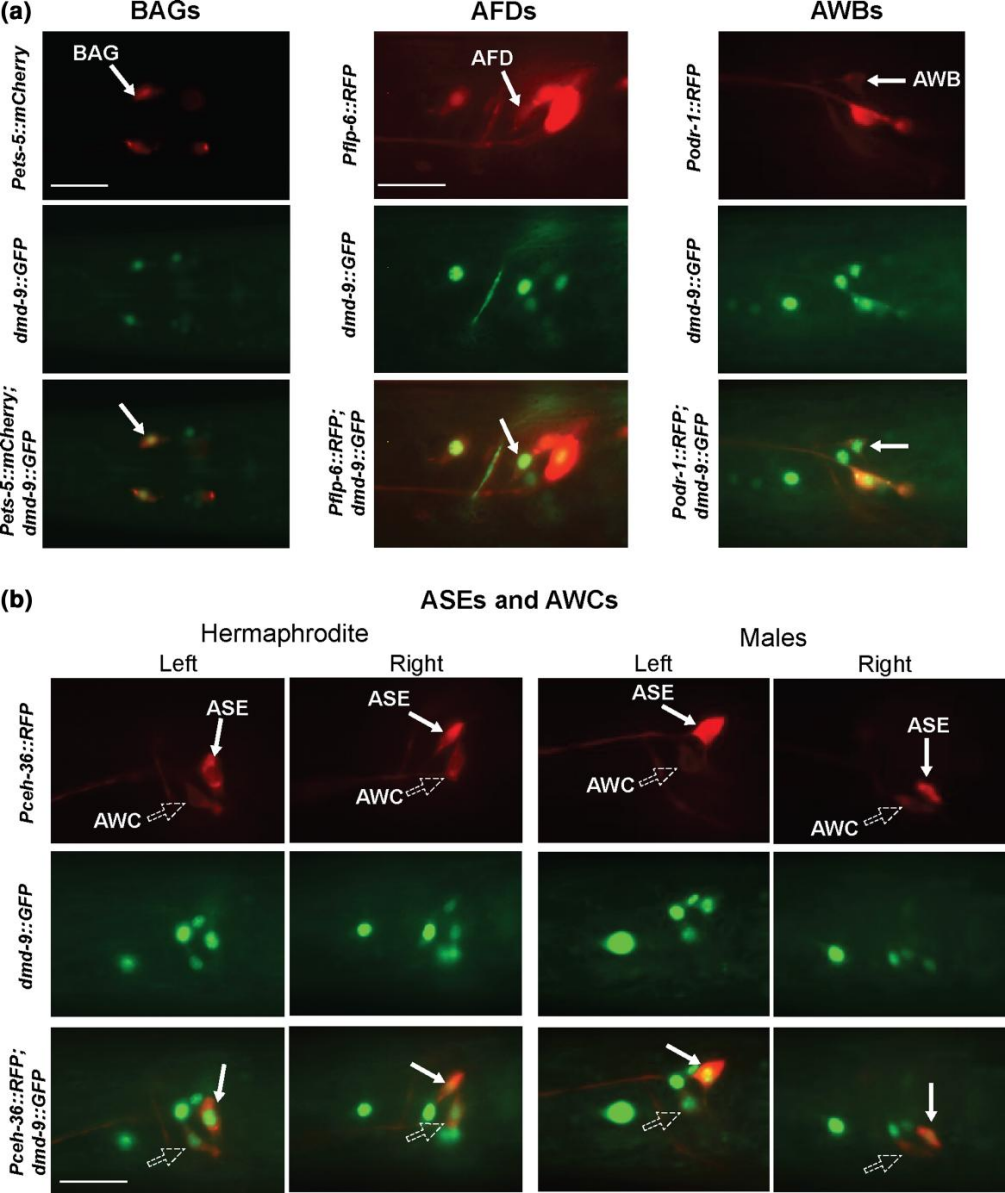
Co-localization of *dmd-9::GFP* and neuron-specific reporters. a) Co-localization of *dmd-9::GFP(rp169)* with neuron-specific markers: BAG (*Pets-5::mCherry*), AFD (*Pflp-6::RFP*) and AWB (*Podr-1::RFP*). Dorsal view. Scale bar = 30 µm. b) Co-localization of *dmd-9::GFP(rp169)* with neuron-specific marker: AWC and ASE (*Pceh-36::RFP*). Lateral view, ventral is down. Scale bar = 20 µm. In all images, anterior is to the left. Strains are: *Pets-5::mCherry(RJP3088); dmd-9::GFP(rp169); Pflp-6::RFP(otIs494); Podr-1::RFP(oyIs44); Pceh-36::RFP(otIs151)*.

We confirmed the identity of DMD-9::GFP-expressing head neurons by co-localization with neuron-specific reporters (Fig. 2a, b). The following reporters were used in this study: *Pets-5::mCherry* (BAGs); *Pflp-6::RFP* (AFDs); *Podr-1::RFP* (AWBs); and *Pceh-36::RFP* (AWC^on^, AWC^off^, ASEL, and ASER; Lanjuin *et al.* 2003; Kim and Li 2004; Koga and Ohshima 2004). In addition, we found that in a small proportion of L4 animals DMD-9::GFP shows asymmetric expression in the ASEs and AWCs neurons (Supplementary Fig. 1).

Taken together, our results confirm the expression of DMD-9 in the neuronal cells identified by single-cell transcriptomic analysis (Taylor *et al.* 2021). We did not detect sexually dimorphic expression for DMD-9::GFP within the nervous system and observed expression in the uterine cells and sperm of hermaphrodites and males.

### DMD-9 cell-autonomously regulates *flp-19* expression in the BAGs

**Fig. 3.**
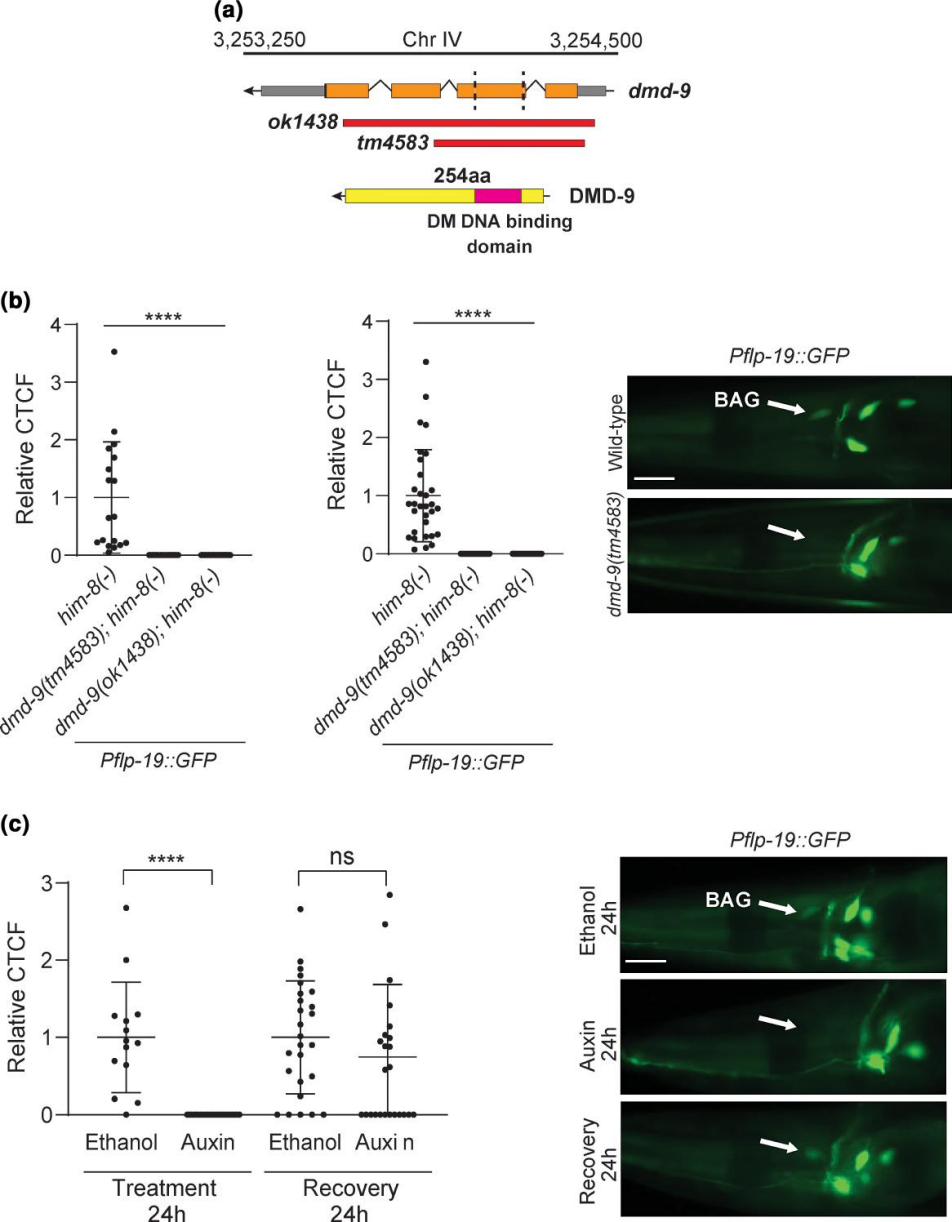
*flp-19* regulation by DMD-9. a) Genomic structure of the *dmd-9* gene, the available deletion mutants and protein structure with identified Doublesex/MAB-3 (DM) DNA binding domain (pink). Dashed lines on the genomic structure indicate the location of the DNA binding domain. b) *Pflp-19::GFP(rpEx1533)* reporter is not expressed in either *dmd-9* deletion allele in hermaphrodites. Scale bar = 20 µm. c) Depletion of DMD-9 from the BAGs causes BAG-specific loss of *Pflp-19::GFP* expression. *Pflp-19::GFP* expression recovered after 24 h of auxin removal. Scale bar = 20 µm. Anterior to the left. *him-8(-)* is *him-8(e1489)*. CTCF = corrected total cell fluorescence. Experiments were performed in 3 biological replications. *n* > 15, ****P-value ≤ 0.0001, ns = not significant. The statistical significance was calculated using one-way ANOVA with Dunnet's correction and unpaired *t*-test. The error bars show SD.

To identify potential DMD-9 functions, we obtained 2 deletion alleles: *dmd-9(tm4583)* and *dmd-9(ok1438)* (Fig. 3a) and crossed these strains into 15 cell fate reporters that are expressed in DMD-9 expressing neurons (Supplementary Table 3). We particularly focused on the BAG neurons by analyzing 6 reporters for the following genes: *flp-13*, *flp-17*, *flp-19*, *gcy-9*, *gcy-31,* and *gcy-33*. To identify sexually dimorphic gene regulation, we analyzed the reporters in both hermaphrodites and males at L4 and adult stages (Supplementary Table 3). Our results show that of all the reporters examined only *Pflp-19::GFP* is lost in the BAG neurons in *dmd-9* mutant hermaphrodites (Fig. 3b). We identified no defects in other *flp-19* expressing hermaphrodite neurons (Fig. 3b). Unfortunately, bright expression of *Pflp-19::GFP* in the male-specific CEM neurons obscured BAG visualization. Finally, we also observed dim *Pgcy-9::GFP* expression in <5% of *dmd-9* mutant animals *(tm4583* and *ok1438)* (Supplementary Table 3).

To determine whether DMD-9 regulates *flp-19* expression cell autonomously, we applied the AID system to deplete DMD-9 specifically in the BAG neurons. We crossed the *dmd-9::GFP::AID* strain into the *Pgcy-9::TIR1* strain that expresses the TIR1 protein only in the BAGs (Pocock *et al.* 2022). We confirmed that DMD-9::GFP is only depleted from BAG neurons and not from other neurons (Supplementary Fig. 2). We then introduced the *dmd-9::GFP::AID; Pgcy-9::TIR1* strain into the *Pflp-19::GFP* reporter and performed auxin depletion. We found that after 24 h of auxin-induced DMD-9::GFP depletion, expression of *Pflp-19::GFP* was undetectable in the BAG neurons (Supplementary Fig. 2b). To reveal if the continual presence of DMD-9::GFP is required for *flp-19* expression we performed an auxin recovery experiment by applying a low concentration of auxin (0.1 mM). The lower auxin concentration also depleted DMD-9::GFP and within 2–3 h after auxin removal DMD-9::GFP was restored (Supplementary Fig. 2c). We repeated this experiment for *Pflp-19::GFP* expression. After 24 h treatment with auxin (0.1 mM) from the L4 stage, *Pflp-19::GFP* expression significantly reduced in the BAGs (Fig. 3c) and *Pflp-19::GFP* expression recovered after 24 h following auxin removal (Fig. 3c). These results show that DMD-9 is cell-autonomously required for continuous *Pflp-19::GFP* expression in the BAGs. However, loss of DMD-9 does not adversely affect the expression of any other neuronal cell fate reporter we analyzed.

### DMD-9 has no detectable behavioral function

We investigated the function of DMD-9 in *C. elegans* behavior by focusing on the behaviors controlled by DMD-9-expressing neurons. For the majority of behaviors, we performed assays in both hermaphrodites and males to identify potential sexually dimorphic regulatory mechanisms controlled by DMD-9.

#### Exploration and mate-searching behavior

**Fig. 4.**
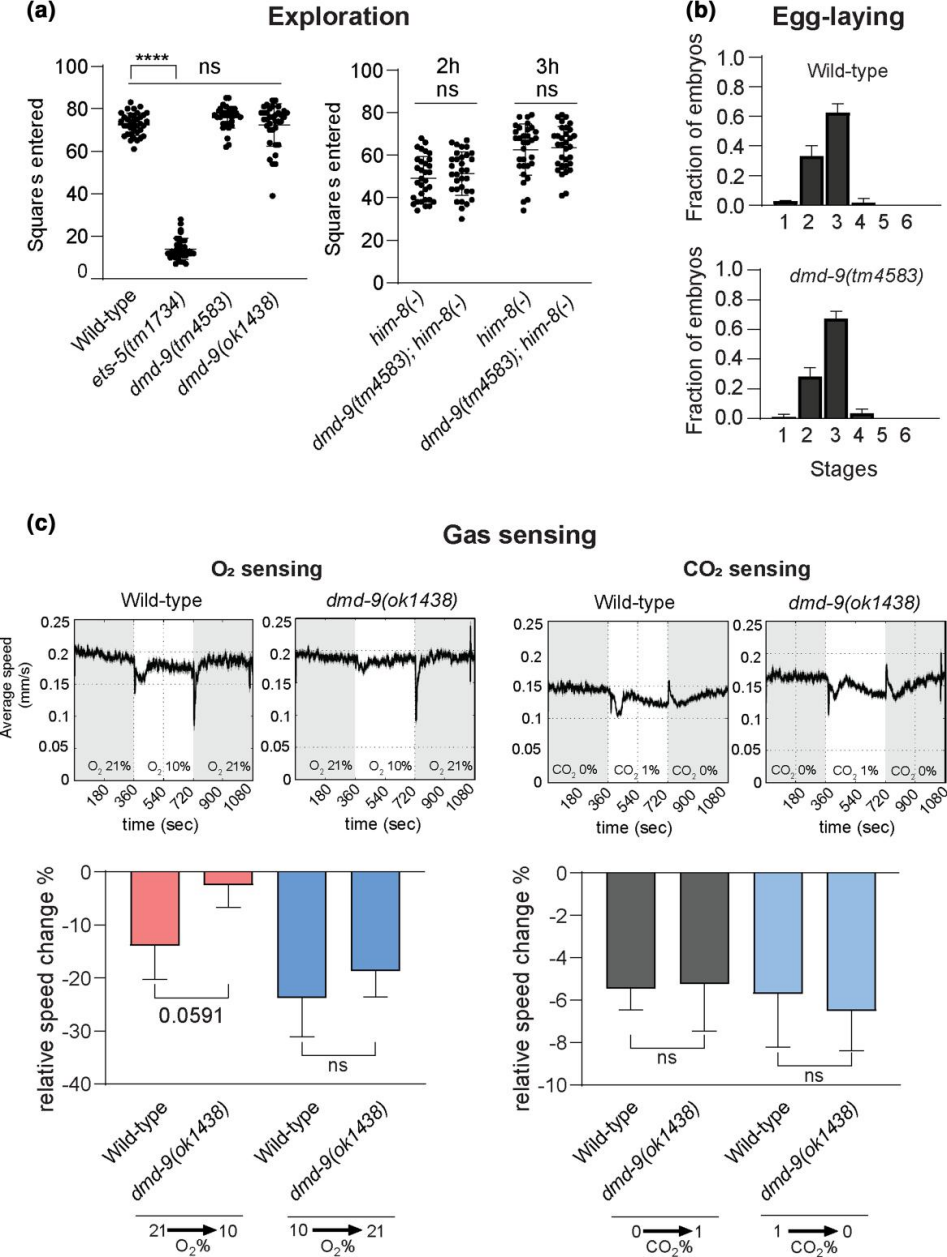
Role of DMD-9 in the BAG-mediated behaviors. a) Exploration behavior of L4 hermaphrodites over 16 h and males for 2 and 3 h. *ets-5(tm1734)* mutant animals used as an exploration-defective control in hermaphrodites. *him-8(-)* = *him-8(e1489)*. b) Egg-laying behavior. The *X* axis show the stage of eggs according to Ringstad and Horvitz (2008). There was no statistical significance in egg-laying behavior between wild-type and *dmd-9(tm4583)* mutant animals. c) Gas sensing behavior. O_2_ and CO_2_ sensing in late L4/young adult hermaphrodites. Experiments were performed in 3 biological replications. For a) *n* > 30, b) *n* > 100, c) *n* > 400. ****P-value ≤ 0.0001, ns = not significant. For a) one-way ANOVA with Dunnet's correction and unpaired *t*-test, For b) Wilcoxon Mann–Whitney rank-sum test, and for c) unpaired *t*-test was used to calculate statistical significance. The error bars show SD.

Exploration is controlled by multiple neurons, including the BAGs (Kniazeva *et al.* 2015; Juozaityte *et al.* 2017). We measured hermaphrodite exploration by tracking animal movement over a 16 h period. In contrast, we assayed male exploration over 2 and 3 h periods. In addition, we assayed male mate-searching (food-leaving) behavior (Lipton *et al.* 2004; Barrios *et al.* 2008, 2012). We used the *dmd-9(tm4583)* allele for these experiments. Our analysis of both hermaphrodites and males shows that DMD-9 is not required for exploration or food-leaving behaviors (Fig. 4a, Supplementary Fig. 3a).

#### Egg-laying

Egg-laying is regulated by multiple neurons, including the BAGs, and tissues such as the vulva and uterus (Trent *et al.* 1983; Schafer 2005; Ringstad and Horvitz 2008). As DMD-9 is expressed in the BAGs and uterine cells we analyzed egg-lying in *dmd-9(tm4583)* mutant animals. Our results did not show any difference in egg-laying behavior between wild-type and *dmd-9(tm4583)* animals (Fig. 4b).

#### Gas sensing behavior

BAG neuron function is required for sensing O_2_ and CO_2_, therefore, we investigated the function of DMD-9 in sensing these gases (Hallem and Sternberg 2008; Zimmer *et al.* 2009). The experiment was performed by analyzing animal motility in response to changes in gas concentration within a sealed chamber (Romanos *et al.* 2015). Animals were exposed to switching between air (79% N_2_ and 21% O_2_) and 10% O_2_ or 1% CO_2_. The results show that *dmd-9(ok1438)* mutants did not show significant defects in O_2_ or CO_2_ sensing (Fig. 4c).

#### Chemosensory behaviors

Chemosensation is regulated by multiple neurons including the AWBs, AWCs, and ASEs (Bargmann and Horvitz 1991; Bargmann *et al.* 1993; Troemel *et al.* 1997). Since all of these neurons express DMD-9, we investigated the role of DMD-9 in regulating chemotaxis in both hermaphrodites and males. We applied high and low concentrations of each chemical and studied animal responses (Supplementary Fig. 3b shows responses to high concentrations of chemicals—low concentration is not shown as results were similar). We analyzed chemotaxis against the repellent 2-nonanone that is sensed by the AWBs (Troemel *et al.* 1997; Supplementary Fig. 3b). We found that *dmd-9(ok1438)* animals responded as wild-type to 2-nonanone (1:10 or 1:100 dilutions). Interestingly, wild-type males were not repelled by 2-nonanone at either concentration, suggesting sex-specific repulsion to this odor (Supplementary Fig. 3b). Tanner *et al*. (2022) identified that males were repelled from undiluted 2-nonanone similar to hermaphrodites. However, males showed delayed food-leaving behavior in the presence of 2-nonanone and food, indicating an impact of 2-nonanone on decision-making. To assess ASE neuron function, we examined chemotaxis toward 1 and 0.25 M of NaCl. Our results also did not show any defects in *dmd-9(ok1438)* mutant animals (Supplementary Fig. 3b). For AWC neuron function, we studied isoamyl alcohol (1:10 and 1:100) and benzaldehyde (1:1,000 and 1:10,000) attraction. *dmd-9(ok1438)* mutant animals exhibited wild-type attraction to these odors (Supplementary Fig. 3b). Taken together, our analysis shows that DMD-9 loss does not impact behaviors regulated by neurons within which it is expressed.

### DMD-9 regulation by neuron fate determination factors

We investigated whether neuronal fate determination factors regulate the expression of DMD-9 in head neurons. We crossed *dmd-9::GFP(rp169)* into 7 fate determination TF mutants: *ets-5(tm1734* or *tm866)* and *egl-13(ku194)* for the BAGs; *che-1(ot866)* for the ASEs; *ttx-1(p767)* for the AFDs; *lim-4(yz12)* for the AWBs; *mls-2(tm252)* and *ceh-37(ok642)* for the AWCs (Sagasti *et al.* 1999; Satterlee *et al.* 2001; Lanjuin *et al.* 2003; Uchida *et al.* 2003; Kim *et al.* 2010; Guillermin *et al.* 2011; Gramstrup Petersen *et al.* 2013). Moreover, we investigated whether DMD-9 regulates the expression of these TFs and whether DMD-9 autoregulates by analyzing the expression of 2 transcriptional GFP reporters driven by *dmd-9* promoters.

**Fig. 5.**
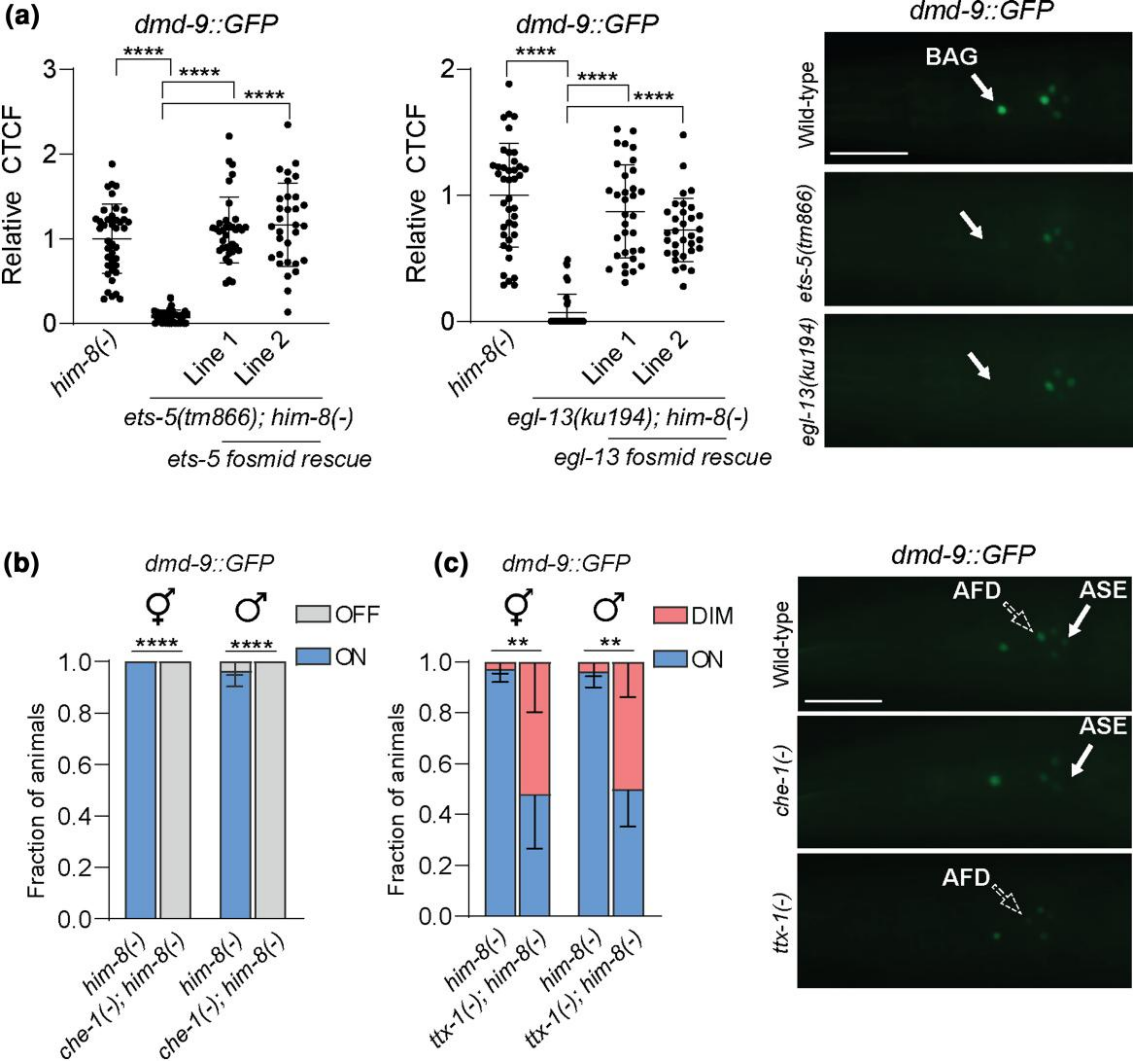
DMD-9 regulation by fate determination tfs. a) *dmd-9::GFP(rp169)* regulation by ETS-5 and EGL-13 TFs in L4 stage hermaphrodites. Comparisons between samples are based the relative expression of GFP to wild-type. Fosmid rescue lines significantly increased the expression of *dmd-9::GFP(rp169)* in mutant animals. Scale bar = 30 µm. b) and c) regulation of *dmd-9::GFP(rp169)* by CHE-1 and TTX-1 TFs. Stacked bar plots show comparisons between the wild-type and mutants at the L4 stage in both hermaphrodites and males. Scale bar = 30 µm. Lateral view, anterior to the left. *him-8(-)* is *him-8(e1489)*; *che-1(-)* is *che-1(ot866)*; *ttx-1(-)* is *ttx-1 (p767)*. CTCF = corrected total cell fluorescence. Experiments were performed in 2 or 3 biological replications. For a) the significance of the results was obtained by one-way ANOVA with Dunnet's correction for the a) and two-way ANOVA analyzes with comparing simple effect within columns for the b) and c). *n* > 30, ****P-value ≤ 0.0001, **P-value ≤ 0.01, ns = not significant. The error bars show SD.

We found that 4 fate-determining TFs regulate DMD-9 expression in different neurons. In the BAG neurons, the ETS-5 and EGL-13 TFs are required for DMD-9::GFP expression (Fig. 5a). Fosmid rescue restored DMD-9::GFP expression in the respective mutants (Fig. 5a). Loss of CHE-1 also caused loss of DMD-9::GFP expression in the ASE neurons in both hermaphrodites and males (Fig. 5b). In the AFD neurons, TTX-1 is partially required for DMD-9::GFP expression, with >50% of AFD neurons exhibiting dim expression at the L4 stage of both sexes; a defect which is exacerbated as animals become adult (Fig. 5c, Supplementary Fig. 4). In contrast, we did not observe any reduction in DMD-9::GFP expression with loss of MLS-2, CEH-37 and LIM-4—TFs required for AWBs and AWCs neuron specification (Supplementary Table 5). Finally, we found that DMD-9 does not regulate the expression of any of the fate determination factors studied, and there is no evidence of DMD-9 autoregulation (Supplementary Table 4). Taken together, our results show that DMD-9 expression is regulated by the ETS-5 and EGL-13 TFs in the BAGs, TTX-1 in the AFDs, and CHE-1 in the ASEs. DMD-9 does not regulate the fate of regulatory TFs or show autoregulation.

### *cis*-Regulatory analysis of the *dmd-9* promoter

**Fig. 6.**
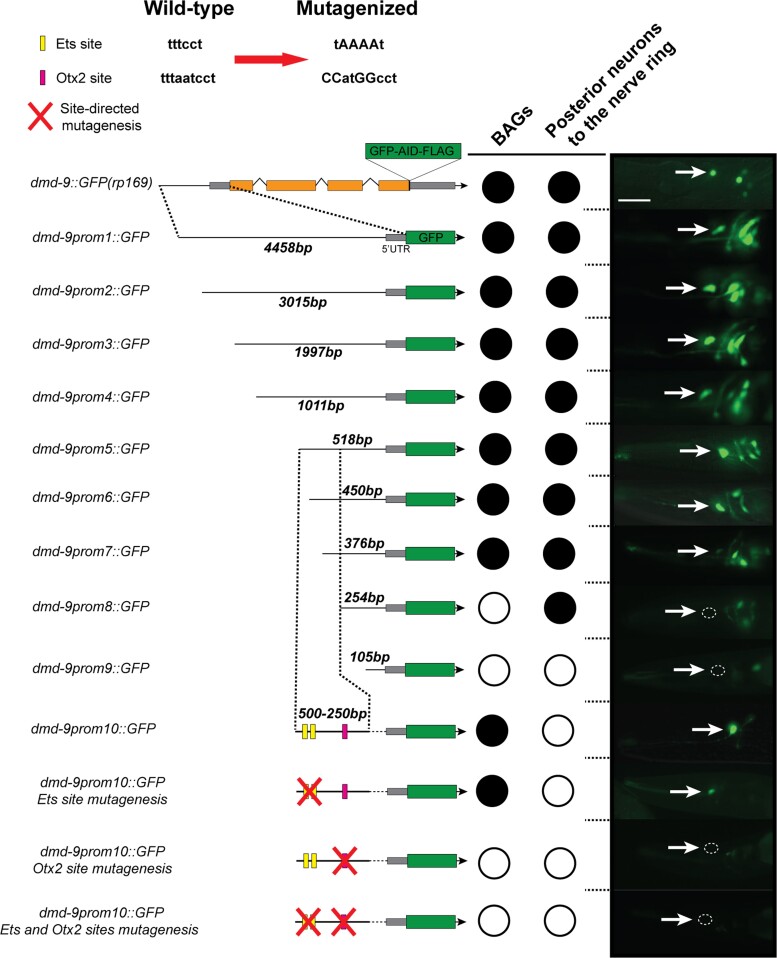
*dmd-9* promoter dissection analysis. We used promoter deletion and mutagenesis to identify regions and elements that regulate *dmd-9* expression in the BAGs and the posterior neurons to the nerve ring. For each constructed reporter, 3 independent lines and more than 20 animals were analyzed. White arrows show BAG neurons location. Lateral view, anterior to the left. Scale bar = 20 µm.

We have shown that DMD-9 is regulated by multiple neuron-specific fate-determining TFs (Fig. 5). Based on these observations, we examined which regulatory elements within the *dmd-9* promoter direct neuron-specific expression. To investigate this, we used a fluorescent reporter driven by a 4.5 kb *dmd-9* promoter that drives GFP in the BAGs, neurons posterior to the nerve ring, and uterine cells (Fig. 6). We truncated this template reporter by PCR to generate 3, 2, 1 kb, 500, 450, 375, 250, and 100 bp versions of the promoter to drive GFP (Fig. 6). Our results show that the 500 bp promoter (*prom5*) is sufficient to drive GFP in the same neurons as the full promoter (4.5 kb). However, the *dmd-9prom8::GFP* (250 bp promoter) was no longer expressed in the BAGs but maintained expression in neurons posterior to the nerve ring. The *dmd-9prom9::GFP* (100 bp promoter) is not expressed in any neurons (Fig. 6). We focused on the regulatory elements in the *dmd-9* promoter that control GFP expression in the BAGs by further dissecting the 500–250 bp region into *dmd-9prom6::GFP* (450 bp) and *dmd-9prom7::GFP* (376 bp). We found that *dmd-9prom7::GFP* drives GFP in the BAG neurons at reduced intensity compared with *dmd-9prom6::GFP*. We constructed a GFP reporter (*dmd-9prom10::GFP*) by cloning the DNA sequence 250–500 bp upstream of the ATG codon of *dmd-9* into the pPD95.75 GFP vector and found that it drives robust GFP expression in the BAG neurons (Fig. 6). Analysis of this sequence in JASPAR (jaspar.genereg.net) identified 2 potential regulatory elements: 2 Ets sites and 1 Otx2 site (Fig. 6). Site-directed mutagenesis of the Ets sites showed no overt effect on the GFP expression in the BAG neurons (Fig. 6). In addition, the *dmd-9prom10::GFP* was expressed in *ets-5(tm1734)* mutant animals (Supplementary Table 6). It is therefore likely that ETS-5 regulates *dmd-9* expression in the BAGs indirectly or in combination with other factors (Fig. 5a). We also mutagenized the Otx2 site alone or in combination with the Ets mutated sites. We found that GFP expression of *dmd-9prom10::GFP* is completely lost in BAG neurons following mutagenesis of the Otx2 site. Potential TFs that may bind to Otx2 elements include the OTX TFs CEH-36, CEH-37, and TTX-1; however, these TFs are not expressed in the BAG neurons (Lanjuin *et al.* 2003). Therefore, we searched for other homeobox TFs expressed in the BAGs that potentially regulate DMD-9 through the Otx2 element. We identified the CEH-23 and CEH-54 homeobox TFs as candidates and introduced the *dmd-9prom10::GFP* construct into *ceh-23(ms23)* and *ceh-54(tm242)* mutants. We found no change in GFP expression in the BAGs compared to wild-type animals (Supplementary Table 6). Taken together, *cis-*regulatory analysis of the *dmd-9* promoter identified a 500 bp upstream region that is sufficient for head neuron expression. We found that at least 1 Otx2 element is required to drive BAG expression; however, the identity of the factors regulating this site is unknown.

## Discussion

The *C. elegans* DMD family encompasses 11 TFs, some of which control sexually dimorphic characteristics (Shen and Hodgkin 1988; Yi *et al.* 2000; Lints and Emmons 2002; Oren-Suissa *et al.* 2016; Serrano-Saiz *et al.* 2017; Bayer *et al.* 2020; Durbeck *et al.* 2021). Here we analyzed another member of this family, DMD-9. According to single-cell transcriptomic data (Taylor *et al.* 2021), *dmd-9* is expressed in multiple sensory neurons and non-neuronal tissues. We studied the expression of DMD-9 by generating a CRISPR-tagged GFP reporter and confirmed the *dmd-9* expression results from single-cell RNA sequencing (Taylor *et al.* 2021). We did not identify sexually dimorphic expression of *dmd-9*; however, expression in the hermaphrodite-specific uterine cells and sperm was detected. DMD-9 may have a specific function in the development of the uterus as expression occurs during a sub-stage of L4 (Fig. 1e). We did not observe any obvious defects in uterine morphology or egg-laying behavior that is partially regulated by uterine cells (Schafer 2005). Another DMD family member, MAB-23 is also expressed in cells related to egg-laying, including the uterus (Lints and Emmons 2002). Therefore, multiple DMD TFs may act redundantly to control the fate and/or function of uterine cells—a concept requiring future study. DMD-9 is also expressed in the sperm of hermaphrodites and males.

According to our analysis, DMD-9 is not broadly involved in nervous system fate determination. We found that DMD-9 cell-autonomously regulates *flp-19*, which encodes an FMRF-like peptide that is expressed in multiple neurons including the BAGs and AWAs. However, the function of FLP-19 is poorly understood. Elevated FLP-19 expression in the context of an *arcp-1* mutant enhances behavioral responses to CO_2_ (Beets *et al.* 2020). FLP-19 is also required for exploration behavior (Juozaityte *et al.* 2017). In our study, *dmd-9* mutants behave similar to wild-type in both CO_2_ sensing and exploration. Therefore, loss of FLP-19 in the BAG neurons in *dmd-9* mutant animals may cause defects that are yet to be identified.

Analyzing behaviors that could potentially be mediated by *dmd-9*-expressing neurons did not reveal any defects compared to wild-type animals. Studies of other members of the DMD family have shown a wide range of phenotypes including sex-specific behavior and morphological defects (Shen and Hodgkin 1988; Yi *et al.* 2000; Oren-Suissa *et al.* 2016). Sex-specific expression patterns of DMD-3, DMD-4, DMD-5, and DMD-11 regulate sex-specific synaptogenesis in these neurons (Oren-Suissa *et al.* 2016; Serrano-Saiz *et al.* 2017; Bayer *et al.* 2020). DMD-5 and DMD-11 also regulate AVG-mediated male mating behavior (Oren-Suissa *et al.* 2016). Regarding sex-shared behavior, DMD-10 regulates ASH-mediated responses to nose touch and high osmolarity (Durbeck *et al.* 2021). These findings suggest that DMD TFs, [except MAB-3 and MAB-23 which show defects in the morphology of the male animals (Shen and Hodgkin 1988; Yi *et al.* 2000; Lints and Emmons 2002)] do not show severe defects in sex-shared phenotypes; however, more studies are required to identify these associations. The lack of behavioral deficits in *dmd-9* mutant animals suggests subtle phenotypes that cannot be identified by current conventional experiments, or alternatively functional redundancy with other genes which compensate for DMD-9 loss. DMD-4, which is expressed in several head neurons and loss of which is lethal (Bayer *et al.* 2020), and DMD-6 which shows pan-neuronal expression (cengen.shinyapps.io/CengenApp/; Taylor *et al.* 2021) are candidate redundantly acting factors. In addition, the role of DMD-9 in AFD-mediated thermosensory behavior needs to be examined.

DMD-9 is expressed in specific head neurons and is independently regulated by fate-determining TFs in each neuron. We found that in the BAGs, AFDs, and AWCs, *dmd-9* is regulated by cell-specific terminal regulatory TFs. In the BAGs, ETS-5 and EGL-13 regulate neuronal fate by controlling the expression of multiple genes such as *flp-17*, *gcy-9*, *gcy-31*, *gcy-33* (Guillermin *et al*. 2011; Gramstrup Petersen *et al*. 2013). TTX-1 regulates gene batteries of the AFD neurons (Satterlee *et al.* 2001), and the fate and functionality of ASE neurons are regulated by CHE-1 (Uchida *et al.* 2003). Noticeably, *dmd-9* regulation by these fate-determining factors is neuron-specific, suggesting cell-specific regulatory mechanisms or the presence of TF-specific *cis-*regulatory elements. In the BAG neurons, we found that at least 1 Otx2 element within 400 bp upstream of the *dmd-9* start codon drives GFP expression. In addition, elements within 250 bp are required for GFP expression in the neurons posterior to the nerve ring. Otx2 elements may be regulated by CEH-36, CEH-37, and TTX-1 Otx homeodomain TFs (Lanjuin *et al.* 2003); however, they are not expressed in the BAG neurons. Searching for other potential regulators through the Otx2 site, we analyzed the CEH-23 and CEH-54 homeodomain TFs that are expressed in the BAG neurons. However, the loss of these TFs did not affect DMD-9 expression in the BAG neurons. ETS-5 is required for *dmd-9* expression in the BAGs but shows no effect on the transcriptional reporter carrying Ets and Otx2 elements. Therefore, ETS-5 and EGL-13 likely regulate DMD-9 expression indirectly through other unknown regulatory factors in the BAG neurons.

### Conclusion

In this study, we characterized the DMD-9 TF in both hermaphrodites and males. We found that FLP-19 expression in the BAGs is cell-autonomously regulated by DMD-9. We also identified the *cis*-regulatory elements and *trans*-acting factors required for DMD-9 expression in specific sensory neurons.

## Supplementary Material

jkac305_Supplementary_Data

## Data Availability

Strains and plasmids are available upon request. The authors affirm that all data necessary for confirming the conclusions of the article are present within the article, figures, and tables. Supplemental material is available at G3 online.

## Notes

*Communicating editor: H. Salz*
