# Supplementary material for: Characterization of the Doublesex/MAB-3 transcription factor DMD-9 in Caenorhabditis elegans
Source: G3 (Bethesda). 2022 Dec 1;13(2):jkac305. doi: 10.1093/g3journal/jkac305 (PMC9911054; doi:10.1093/g3journal/jkac305)
Supplement: jkac305_Supplementary_Data [file jkac305_supplementary_data.zip › Supplemental_Figure_Legends_G3-2022-403934.docx]

# Supplementary Figure Legends

**Fig S1.** Analysis of the *dmd-9::GFP(rp169)* expression in the left and right AWC and ASE neurons. Experiments were performed in 3 biological replications. n >40. We used two-way ANOVA analysis by comparing simple effect within columns to analyze significance of the results. The error bars show SD.

**Fig S2.** Depletion of DMD-9::GFP protein using the AID system.

1. All the animals lost DMD-9::GFP expression in the BAG neurons of animals carrying the *Pgcy-9::TIR1* transgene that were exposed to auxin.
2. DMD-9::GFP protein is depleted from the BAGs after 2h of auxin treatment. *Pflp-19::GFP* expression is also undetectable following 24h auxin treatment. Scale bar = 20 μm.
3. Auxin treatment and recovery of DMD-9. Animals were exposed to auxin (0.1mM) for 2h and confirmed for depletion of *dmd-9* expression in the BAGs. A portion of animals were transferred to NGM plates and incubated for 3h and then examined for recovery of *dmd-9* expression.

White arrows show the BAG neurons. CTCF = Corrected Total Cell Fluorescence. Experiments were performed in 3 biological replications. *n* >15*, ***** = *P*-value ≤ 0.0001, ns = not significant. The unpaired t-student test was used to obtain statistical significance. The error bars show SD.

**Fig S3.** *dmd-9* behavioral analysis.

1. Mate searching behavior in both adult males and hermaphrodites. *pdfr-1(ok3425)* mutant used as a male leaving-deficient control.
2. Chemosensory behaviors regulated by AWB, AWC and ASE neurons. 2-butanone, benzaldehyde, isoamyl alcohol, and NaCl were used as attractant chemicals, while 2-nonanone was used as repellent. For these experiments, positive controls were as follows: *odr-3(n2150)* for 2-butanone, benzaldehyde, isoamyl alcohol, and 2-nonanone; *che-1(ot866)* for NaCl (not shown).

*him-8(-)* is *him-8(e1489)*. Experiments were performed in 5 biological replications. For the mate-searching behavior *n* > 60. For the chemosensory behaviors *n* >300*, ***** = *P-value* ≤ 0.0001, ns = not significant. For **A** the Gehan-Breslow-Wilcoxon test and For **B** unpaired t-test were applied to calculate statistical significance. The error bars show SD.

**Fig S4.** *dmd-9::GFP(rp169)* regulation by CHE-1 and TTX-1 TFs. Experiments were performed in 2 biological replications. *him-8(-)* is *him-8(e1489)*; *che-1(-)* is *che-1(ot866)*; *ttx-1(-)* is *ttx-1 (p767)*. The significance of the results was obtained by two-way ANOVA analysis by comparing simple effect within column analysis. *n* >30*, ***** = *P-value* ≤ 0.0001, ***** = *P-value* ≤ 0.001, ns = not significant. The error bars show SD.
