# Supplementary material for: Characterization of the Doublesex/MAB-3 transcription factor DMD-9 in Caenorhabditis elegans
Source: G3 (Bethesda). 2022 Dec 1;13(2):jkac305. doi: 10.1093/g3journal/jkac305 (PMC9911054; doi:10.1093/g3journal/jkac305)
Supplement: jkac305_Supplementary_Data [file jkac305_supplementary_data.zip › Table_S1_G3-2022-403934.docx]

**Table S1. Strains used in this study.**

| **Strain name** | **Genotype** |
| --- | --- |
| N2 Bristol | *C. elegans* wild isolate. |
| RJP4442 | *him-8(e1489) IV* |
| RJP4622 | *dmd-9(tm4583) IV* |
| RJP4621 | *dmd-9(ok1438) IV* |
| RJP4623 | *dmd-9(tm4583) him-8(e1489) IV* |
| RJP4652 | *dmd-9(ok1438) him-8(e1489) IV* |
| RJP235 | *ets-5(tm1734) X* |
| RJP5357 | *rp169[dmd-9::GFP-AID-TEV-FLAG] IV* |
| RJP5174 | *rp169[dmd-9::GFP-AID-TEV-FLAG] him-8(e1489) IV* |
| RJP5452 | *him-8(e1489) IV; kyIs104[str-1p::GFP] X* |
| RJP5480 | *dmd-9(ok1438) him-8(e1489) IV; kyIs104[str-1p::GFP] X* |
| RJP5045 | *him-8(e1489) IV; oyIs44 [odr-1::RFP + lin-15(+)]* |
| RJP5057 | *dmd-9(tm4583) him-8(e1489) IV; oyIs44 [odr-1::RFP + lin-15(+)] V* |
| RJP5362 | *pyIs500[ofm-1p::GFP + odr-1p::DsRed + odr-3p::GFP::egl-4]; him-8(e1489) IV* |
| RJP5411 | *pyIs500[ofm-1p::GFP + odr-1p::DsRed + odr-3p::GFP::egl-4]; dmd-9(ok1438) him-8(e1489) IV* |
| RJP5364 | *kyIs140[str-2::GFP + lin-15(+)] I; him-8(e1489) IV* |
| RJP5410 | *kyIs140[str-2::GFP + lin-15(+)] I; dmd-9(ok1438) him-8(e1489) IV* |
| RJP5414 | *kyIs408[srsx-3::GFP; str-2::dsRed2; elt-2::GFP]; him-8(e1489) IV* |
| RJP5415 | *kyIs408[srsx-3::GFP; str-2::dsRed2; elt-2::GFP]; dmd-9(ok1438) him-8(e1489) IV* |
| RJP5365 | *him-8(e1489) IV; ntIs1[gcy-5p::GFP + lin-15(+)] V* |
| RJP5417 | *dmd-9(ok1438) him-8(e1489) IV; [gcy-5p::GFP + lin-15(+)] V* |
| RJP5363 | *him-8(e1489) IV; otIs586[gcy-6(fosmid)::SL2::NLS::GFP + ttx-3p::mCherry] X* |
| RJP5406 | *dmd-9(ok1438) him-8(e1489) IV; otIs586[gcy-6(fosmid)::SL2::NLS::GFP + ttx-3p::mCherry] X* |
| RJP5485 | *otIs4[gcy-7::GFP]; him-8(e1489) IV* |
| RJP5479 | *otIs4[gcy-7::GFP]; dmd-9(ok1438) him-8(e1489) IV* |
| RJP5453 | *him-8(e1489) IV; oyIs18[gcy-8::GFP] X* |
| RJP5484 | *dmd-9(ok1438) him-8(e1489) IV; oyIs18[gcy-8::GFP] X* |
| RJP4640 | *ynIs37[flp-13::GFP] III;him-8(e1489) IV* |
| RJP4695 | *ynIs37[flp-13::GFP] III; dmd-9(tm4583) him-8(e1489) IV* |
| RJP4638 | *ynIs64[flp-17::GFP] I;him-8(e1489) IV* |
| RJP4693 | *ynIs64[flp-17::GFP] I; dmd-9(tm4583) him-8(e1489) IV* |
| RJP4929 | *rpEx1533[PCR 5kb promoter flp-19::GFP + ttx-3::mcherry]; him-8(e1489) IV* |
| RJP4858 | *rpEx1533[PCR 5kb promoter flp-19::GFP + ttx-3::mcherry]; dmd-9(tm4583) him-8(e1489) IV* |
| RJP4984 | *rpEx1533[PCR 5kb promoter flp-19::GFP + ttx-3::mcherry]; dmd-9(ok1438) him-8(e1489) IV* |
| RJP4512 | *wzIs112[gcy-9::rfp]; him-8(e1489) IV* |
| RJP4690 | *wzIs112[gcy-9::rfp]; dmd-9(tm4583) him-8(e1489)IV* |
| RJP4724 | *wzIs112[gcy-9::rfp]; dmd-9(ok1438) him-8(e1489) IV* |
| RJP5280 | *rpIs29[gcy-31::mCherry; elt-2::gfp]; him-8(e1489) IV* |
| RJP4704 | *rpIs29[gcy-31::mCherry; elt-2::gfp]; dmd-9(tm4583) him-8(e1489) IV* |
| RJP4511 | *rpEx274[pegl-13(prom1)::mCherry]; rpIs7[pgcy-33::gfp]; him-8(e1489) IV* |
| RJP4705 | *rpEx274[pegl-13(prom1)::mCherry]; rpIs7[pgcy-33::gfp]; dmd-9(tm4583) him-8(e1489) IV* |
| RJP4462 | *him-8(e1489) IV; nu1646[ets-5::GFP] X* |
| RJP4681 | *dmd-9(tm4583) him-8(e1489) IV; nu1646[ets-5::GFP] X* |
| RJP5346 | *him-8(e1489) IV; kuIs29[egl-13p::GFP + unc-119(+)] V* |
| RJP5374 | *dmd-9(4583) him-8(e1489) IV; kuIs29[egl-13p::GFP + unc-119(+)] V* |
| RJP5511 | *ot856[che-1::gfp] I; him-8(e1489) IV* |
| RJP5510 | *ot856[che-1::gfp] I; dmd-9(ok1438) him-8(e1489) IV* |
| RJP5505 | *wgIs681[lim-4::TY1::EGFP::3xFLAG + unc-119(+)] III; him-8(e1489) IV* |
| RJP5504 | *wgIs681[lim-4::TY1::EGFP::3xFLAG + unc-119(+)] III; dmd-9(ok1438) IV; him-8(e1489) IV* |
| RJP5507 | *him-8(e1489) IV; wgIs645[mls-2::TY1::EGFP::3xFLAG + unc-119(+)] X* |
| RJP5506 | *dmd-9(ok1438) him-8(e1489) IV; wgIs645[mls-2::TY1::EGFP::3xFLAG + unc-119(+)] X* |
| RJP5509 | *him-8(e1489) IV; ot1023[ceh-37::GFP::FLAG] X* |
| RJP5508 | *dmd-9(ok1438) him-8(e1489) IV; ot1023[ceh-37::GFP::FLAG] X* |
| RJP5525 | *rpEx2227[Pdmd-9::GFP]* |
| RJP5533 | *rpEx2227[Pdmd-9::GFP]; dmd-9(ok1438) him-8(e1489) IV* |
| RJP5418 | *rp169[dmd-9::GFP-AID-TEV-FLAG] him-8(e1489) IV; mls-2(tm252) X* |
| RJP5548 | *rp169[dmd-9::GFP-AID-TEV-FLAG] him-8(e1489) IV; ceh-37(ok642) X* |
| RJP5549 | *rp169[dmd-9::GFP-AID-TEV-FLAG] him-8(e1489) IV; lim-4(yz12) X* |
| RJP5261 | *him-8(e1489) IV; oyIs44[odr-1::RFP] V* |
| RJP5251 | *rp169[dmd-9::GFP-AID-TEV-FLAG] him-8(e1489) IV; oyIs44[odr-1::RFP] V* |
| RJP5343 | *otIs494[flp-6(fosmid)::sl2::1xNLS::mChOpti]; him-8(e1489) IV* |
| RJP5316 | *otIs494[flp-6(fosmid)::sl2::1xNLS::mChOpti]; rp169[dmd-9::GFP-AID-TEV-FLAG] him-8(e1489) IV* |
| RJP4490 | *rpEx1523[Pets-5::mCherry]; him-8(e1489) IV* |
| RJP5279 | *rpEx1523[Pets-5::mCherry]; rp169[dmd-9::GFP-AID-TEV-FLAG] him-8(e1489) IV* |
| RJP4948 | *otIs151[ceh-36p::RFP + rol-6(su1006)]; him-8(e1489) IV* |
| RJP5300 | *otIs151[ceh-36p::RFP + rol-6(su1006)]; rp169[dmd-9::GFP-AID-TEV-FLAG] him-8(e1489) IV* |
| RJP5264 | *rpSi1[pgcy-9::TIR1::F2A::mTagBFP2::NLS::AID::tbb-2 3’UTR] II; rp169[dmd-9::GFP-AID-TEV-FLAG] IV* |
| RJP5292 | *rpSi1[pgcy-9::TIR1::F2A::mTagBFP2::NLS::AID::tbb-2 3’UTR] II;  rpEx1533[Pflp-19::GFP + ttx-3::mcherry]; rp169[dmd-9::GFP-AID-TEV-FLAG] him-8(e1489)IV* |
| RJP5344 | *rp169[dmd-9::GFP-AID-TEV-FLAG] him-8(e1489) IV; ets-5(tm866) X* |
| RJP5324 | *rp169[dmd-9::GFP-AID-TEV-FLAG] him-8(e1489) IV; ets-5(tm1734) X* |
| RJP5412 | *rp169[dmd-9::GFP-AID-TEV-FLAG] him-8(e1489) IV; ets-5(tm1734) X; ets-5 fosmid rescue* |
| RJP5413 |  |
| RJP5481 | *rp169[dmd-9::GFP-AID-TEV-FLAG] him-8(e1489) IV; egl-13(ku194) X* |
| RJP5682 | *rp169[dmd-9::GFP-AID-TEV-FLAG] him-8(e1489) IV; egl-13(ku194) X; egl-13 fosmid rescue* |
| RJP5683 |  |
| RJP5607 | *che-1(ot866) I; rp169[dmd-9::GFP-AID-TEV-FLAG] him-8(e1489) IV* |
| RJP5606 | *rp169[dmd-9::GFP-AID-TEV-FLAG] him-8(e1489) IV; ttx-1(p767) V* |
| RJP4767 | *pdfr-1(ok3425) III; him-8(e1489) IV* |
| RJP5522 | *rpEx2226[dmd-9prom1::GFP]* |
| RJP5530 | *rpEx2232[dmd-9prom2::GFP]* |
| RJP5528 | *rpEx2230[dmd-9prom3::GFP]* |
| RJP5526 | *rpEx2228[dmd-9prom4::GFP]* |
| RJP5539 | *rpEx2236[dmd-9prom5::GFP]* |
| RJP5584 | *rpEx2260[dmd-9prom6::GFP]* |
| RJP5586 | *rpEx2262[dmd-9prom7::GFP]* |
| RJP5543 | *rpEx2240[dmd-9prom8::GFP]* |
| RJP5545 | *rpEx2242[dmd-9prom9::GFP]* |
| RJP5592 | *rpEx2264[dmd-9prom10::GFP]* |
| RJP5672 | *rpEx2264[dmd-9prom10::GFP]; ceh-23(ms23) III* |
| RJP5674 | *rpEx2264[dmd-9prom10::GFP]; ceh-54(tm242) X* |
| RJP5611 | *rpEx2264[dmd-9prom10::GFP]; ets-5(tm173) X* |
| RJP5615 | *rpEx2268[dmd-9prom10::GFP ΔEts1/Ets2]* |
| RJP5628 | *rpEx2279[dmd-9prom10::GFP ΔOtx2]* |
| RJP5626 | *rpEx2277[dmd-9prom10::GFP ΔEts1/Ets2/Otx2]* |
