# Supplementary material for: Characterization of the Doublesex/MAB-3 transcription factor DMD-9 in Caenorhabditis elegans
Source: G3 (Bethesda). 2022 Dec 1;13(2):jkac305. doi: 10.1093/g3journal/jkac305 (PMC9911054; doi:10.1093/g3journal/jkac305)
Supplement: jkac305_Supplementary_Data [file jkac305_supplementary_data.zip › Table_S2_G3-2022-403934.docx]

**Table S2.** **Oligos used for GFP knock-in to *dmd-9* using CRISPR-Cas9.**

| **Oligos** | **Sequence** | **Product size (bp)** |
| --- | --- | --- |
| Alt-R CRISPR-Cas9 crRNA | AAGGAACTGATAGACCGTTC AGG |  |
| *dmd-9* F ULTRAMER-GFP-AID-TEV-FLAG | AACCCAACCAACGTCTCTCCAGCACTCATCTCCTTCCTCCTCCAGCCAACCCAGCAGCCATCATTCGAGCCATCCACCATGTCTCTAGCACCACCAGTCTTGTTCCCCACCTTCCTGAACGGTGGCGGTGGATCGGGAGG | 1476 |
| *dmd-9* R ULTRAMER-GFP-AID-TEV-FLAG | AAAAAATCCCCATTGGGGAATACGGTGGGTGTGTGTCGGTGTGACCATACTAGTCACTTGACAATGAAACAATGAGCACGGGTTAGTAGCTCACACGAGAGGGAAGGAACTGATAGACCCTTGTCATCGTCATCCTTGT | 1476 |
| 5' LINKER-GFP-AID-TEV-FLAG F | GGTGGCGGTGGATCGGG | 1236 |
| 5' LINKER-GFP-AID-TEV-FLAG R | CTTGTCATCGTCATCCTTGTAATC | 1236 |
