# Supplementary material for: Characterization of the Doublesex/MAB-3 transcription factor DMD-9 in Caenorhabditis elegans
Source: G3 (Bethesda). 2022 Dec 1;13(2):jkac305. doi: 10.1093/g3journal/jkac305 (PMC9911054; doi:10.1093/g3journal/jkac305)
Supplement: jkac305_Supplementary_Data [file jkac305_supplementary_data.zip › Table_S3_G3-2022-403934.docx]

**Table S3. Neuron fate reporters used in this study and the impact of the DMD-9 TF on their expression in L4 and adult hermaphrodites and males.** Numbers show the percentage of animals expressing the reporter. n > 50.

| **Neuron** | **Genotype** | | **Hermaphrodite** | | **Male** | |
| --- | --- | --- | --- | --- | --- | --- |
|  | Reporter | Mutants | **L4** | **Adult** | **L4** | **Adult** |
| **AWB** | *Pstr-1::GFP*  *(kyIs104)* | *him-8(e1489)* | 100 | 100 | 100 | 100 |
|  |  | *dmd-9(ok1438); him-8(e1489)* | 100 | 100 | 100 | 100 |
| **AWB/**  **AWC** | *Podr-1::RFP*  *(oyIs44)* | *him-8(e1489)* | 100 | 100 | 100 | 100 |
|  |  | *dmd-9(ok1438); him-8(e1489)* | 100 | 100 | 100 | 100 |
|  | *Podr-3::GFP*  *(pyIs500)* | *him-8(e1489)* | 100 | 100 | 100 | 100 |
|  |  | *dmd-9(ok1438); him-8(e1489)* | 100 | 100 | 100 | 100 |
| **AWC** | *str-2::GFP*  *(kyIs140)* | *him-8(e1489)* | 100 | 100 | 100 | 100 |
|  |  | *dmd-9(ok1438); him-8(e1489)* | 100 | 100 | 100 | 100 |
|  | *srsx-3::GFP*  *(kyIs408)* | *him-8(e1489)* | 100 | 100 | 100 | 100 |
|  |  | *dmd-9(ok1438); him-8(e1489)* | 100 | 100 | 100 | 100 |
| **ASE** | *Pgcy-5::GFP*  *(ntIs1)* | *him-8(e1489)* | 100 | 100 | 100 | 100 |
|  |  | *dmd-9(ok1438); him-8(e1489)* | 100 | 100 | 100 | 100 |
|  | *gcy-6::GFP*  *(otIs586)* | *him-8(e1489)* | 100 | 100 | 100 | 100 |
|  |  | *dmd-9(ok1438); him-8(e1489)* | 100 | 100 | 100 | 100 |
| **ASE** | *Pgcy-7::GFP*  *(otIs4)* | *him-8(e1489)* | 100 | 100 | 100 | 100 |
|  |  | *dmd-9(ok1438); him-8(e1489)* | 100 | 100 | 100 | 100 |
| **AFD** | *gcy-8::GFP*  *(oyIs18)* | *him-8(e1489)* | 100 | 100 | 100 | 100 |
|  |  | *dmd-9(ok1438); him-8(e1489)* | 100 | 100 | 100 | 100 |
| **BAG** | *Pflp-13::GFP*  *(ynIs37)* | *him-8(e1489)* | 88 | 83 | 98 | 98 |
|  |  | *dmd-9(tm4583); him-8(e1489)* | 93 | 81 | 100 | 94 |
|  | *Pflp-17::GFP*  *(ynIs64)* | *him-8(e1489)* | 100 | 100 | 100 | 100 |
|  |  | *dmd-9(tm4583); him-8(e1489)* | 100 | 100 | 100 | 100 |
|  | *Pflp-19::GFP*  *(RJP3112)* | *him-8(e1489)* | 100 | 100 | 100 | 100 |
|  |  | *dmd-9(tm4583); him-8(e1489)* | 0 | 0 | 0 | 0 |
|  |  | *dmd-9(ok1438); him-8(e1489)* | 0 | 0 | 0 | 0 |
|  | *Pgcy-9::GFP*  *(wzIs112)* | *him-8(e1489)* | 100 | 100 | 100 | 100 |
|  |  | *dmd-9(tm4583); him-8(e1489)* | 97 | 96 | 96 | 100 |
|  | *Pgcy-31::GFP*  *(rpIs29)* | *him-8(e1489)* | 100 | 100 | 100 | 100 |
|  |  | *dmd-9(tm4583); him-8(e1489)* | 100 | 100 | 100 | 100 |
|  | *Pgcy-33::GFP*  *(RJP602)* | *him-8(e1489)* | 98 | 100 | 98 | 100 |
|  |  | *dmd-9(tm4583); him-8(e1489)* | 100 | 100 | 100 | 100 |
