# Supplementary material for: Characterization of the Doublesex/MAB-3 transcription factor DMD-9 in Caenorhabditis elegans
Source: G3 (Bethesda). 2022 Dec 1;13(2):jkac305. doi: 10.1093/g3journal/jkac305 (PMC9911054; doi:10.1093/g3journal/jkac305)
Supplement: jkac305_Supplementary_Data [file jkac305_supplementary_data.zip › Table_S4_G3-2022-403934.docx]

**Table S4. DMD-9 TF regulation of neuron fate determining TFs**. Numbers show the percentage of animals expressing the reporter. n > 50.

| **Neuron** | **Genotype** | | **Hermaphrodite** | | **Male** | |
| --- | --- | --- | --- | --- | --- | --- |
|  | Reporter | Mutants | **L4** | **Adult** | **L4** | **Adult** |
| **BAG** | *ets-5::GFP*  *(nu1646)* | *him-8(e1489)* | 100 | 100 | 100 | 100 |
|  |  | *dmd-9(tm4583); him-8(e1489)* | 100 | 100 | 100 | 100 |
|  | *egl-13::GFP*  *(kuIs29)* | *him-8(e1489)* | 100 | 100 | 100 | 100 |
|  |  | *dmd-9(ok1438); him-8(e1489)* | 100 | 100 | 100 | 100 |
| **ASE** | *che-1::GFP*  *(ot856)* | *him-8(e1489)* | 100 | 100 | 100 | 100 |
|  |  | *dmd-9(ok1438); him-8(e1489)* | 100 | 100 | 100 | 100 |
| **AWB** | *lim-4::GFP*  *(wgIs681)* | *him-8(e1489)* | 100 | 100 | 100 | 100 |
|  |  | *dmd-9(ok1438); him-8(e1489)* | 100 | 100 | 100 | 100 |
| **AWC/ASE** | *mls-2::GFP*  *(wgIs645)* | *him-8(e1489)* | 100 | 100 | 100 | 100 |
|  |  | *dmd-9(ok1438); him-8(e1489)* | 100 | 100 | 100 | 100 |
| **AWC/ASE/**  **AWB** | *ceh-37::GFP*  *(ot1023)* | *him-8(e1489)* | 100 | 100 | 100 | 100 |
|  |  | *dmd-9(ok1438); him-8(e1489)* | 100 | 100 | 100 | 100 |
| **BAG/AFD/**  **AWC/**  **ASE/AWB** | *rpEx2226[Pdmd-9::GFP]* | *N2* | 100 | 100 | - | - |
|  |  | *dmd-9(ok1438); him-8(e1489)* | 100 | 100 | 100 | 100 |
