# Supplementary material for: Characterization of the Doublesex/MAB-3 transcription factor DMD-9 in Caenorhabditis elegans
Source: G3 (Bethesda). 2022 Dec 1;13(2):jkac305. doi: 10.1093/g3journal/jkac305 (PMC9911054; doi:10.1093/g3journal/jkac305)
Supplement: jkac305_Supplementary_Data [file jkac305_supplementary_data.zip › Table_S5_G3-2022-403934.docx]

**Table S5. Role of neuron fate determining TFs on *dmd-9* regulation.** Numbers show the percentage of animals expressing the reporter. n > 50.

| **Neuron** | **Genotype** | | **Hermaphrodite** | | **Male** | |
| --- | --- | --- | --- | --- | --- | --- |
|  | Reporter | Mutants | **L4** | **Adult** | **L4** | **Adult** |
| **AWC*** | *dmd-9::GFP(rp169)* | *him-8(e1489)* | 97 | 95 | 92 | 100 |
|  |  | *mls-2(tm252);*  *him-8(e1489)* | 95 | 100 | 97 | 100 |
|  | *dmd-9::GFP(rp169)* | *ceh-37(ok642);*  *him-8(e1489)* | 93 | 98 | 98 | 93 |
| **AWB** | *dmd-9::GFP(rp169)* | *him-8(e1489)* | 100 | 100 | 100 | 100 |
|  |  | *lim-4(yz12);*  *him-8(e1489)* | 100 | 100 | 100 | 100 |

*: average of AWC left and right.
