# Supplementary material for: Characterization of the Doublesex/MAB-3 transcription factor DMD-9 in Caenorhabditis elegans
Source: G3 (Bethesda). 2022 Dec 1;13(2):jkac305. doi: 10.1093/g3journal/jkac305 (PMC9911054; doi:10.1093/g3journal/jkac305)
Supplement: jkac305_Supplementary_Data [file jkac305_supplementary_data.zip › Table_S6_G3-2022-403934.docx]

**Table S6. Role of TFs on the regulation of Ets and Otx2 *cis*-regulatory elements.** n > 20.

| **Strains** | **Line 1** | | | **Line 2** | | |
| --- | --- | --- | --- | --- | --- | --- |
|  | **ON** | **dim** | **OFF** | **ON** | **dim** | **OFF** |
| *Wild-type* | 0.41 | 0.13 | 0.44 | 0.33 | 0.08 | 0.57 |
| *ets-5(tm1734)* | 0.33 | 0.13 | 0.52 | 0.32 | 0.12 | 0.55 |
| *ceh-23(ms23)* | 0.34 | 0.27 | 0.37 | 0.26 | 0.2 | 0.53 |
| *ceh-54(tm242)* | 0.33 | 0.11 | 0.55 | 0.31 | 0.15 | 0.53 |
