# Supplementary figures and images for: Characterization of the Doublesex/MAB-3 transcription factor DMD-9 in Caenorhabditis elegans
Source: G3 (Bethesda). 2022 Dec 1;13(2):jkac305. doi: 10.1093/g3journal/jkac305 (PMC9911054; doi:10.1093/g3journal/jkac305)

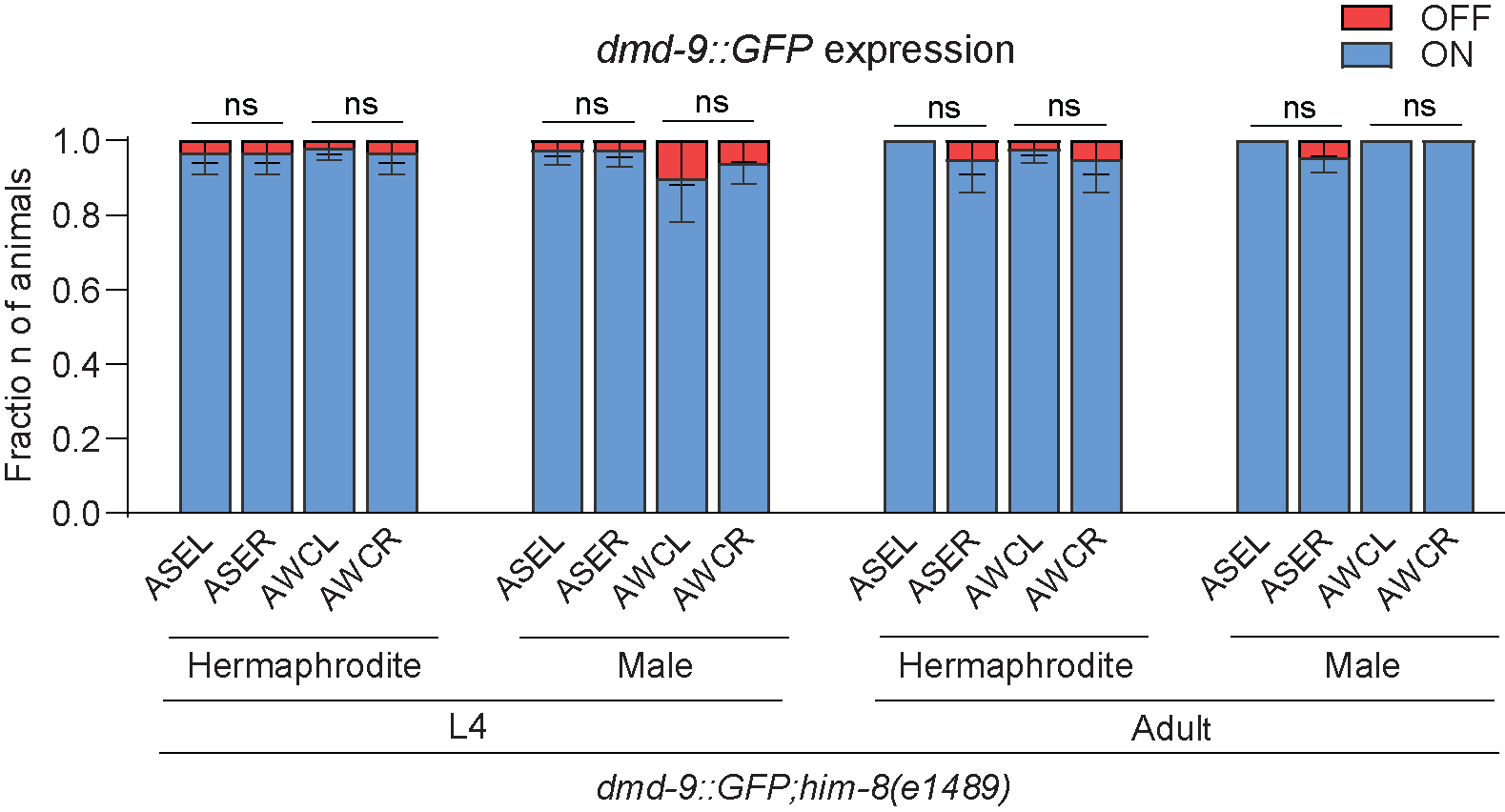

Supplement: jkac305_Supplementary_Data [file jkac305_supplementary_data.zip › Figure_S1_G3-2022-403934.tif]

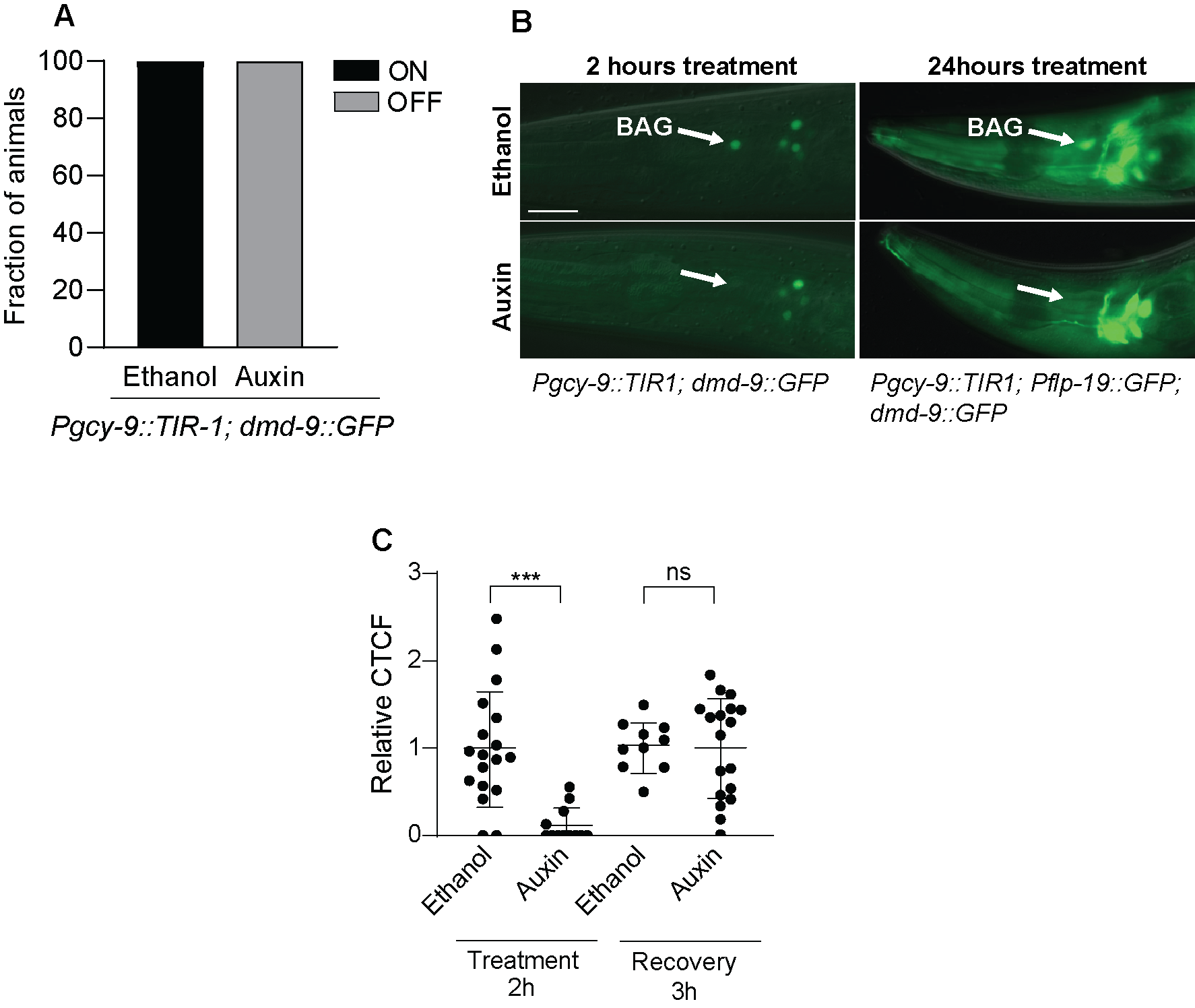

Supplement: jkac305_Supplementary_Data [file jkac305_supplementary_data.zip › Figure_S2_G3-2022-403934.tif]

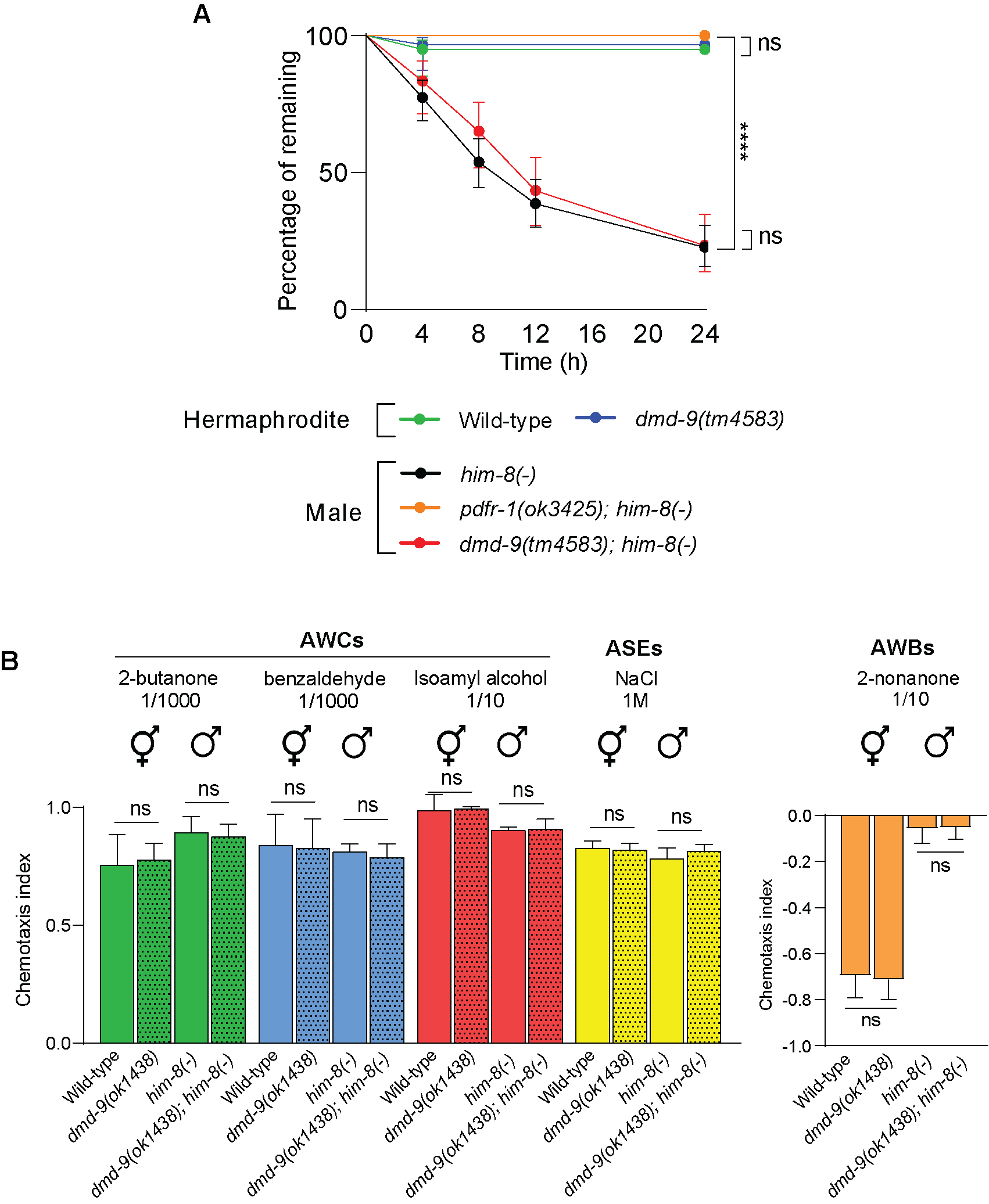

Supplement: jkac305_Supplementary_Data [file jkac305_supplementary_data.zip › Figure_S3_G3-2022-403934.tif]

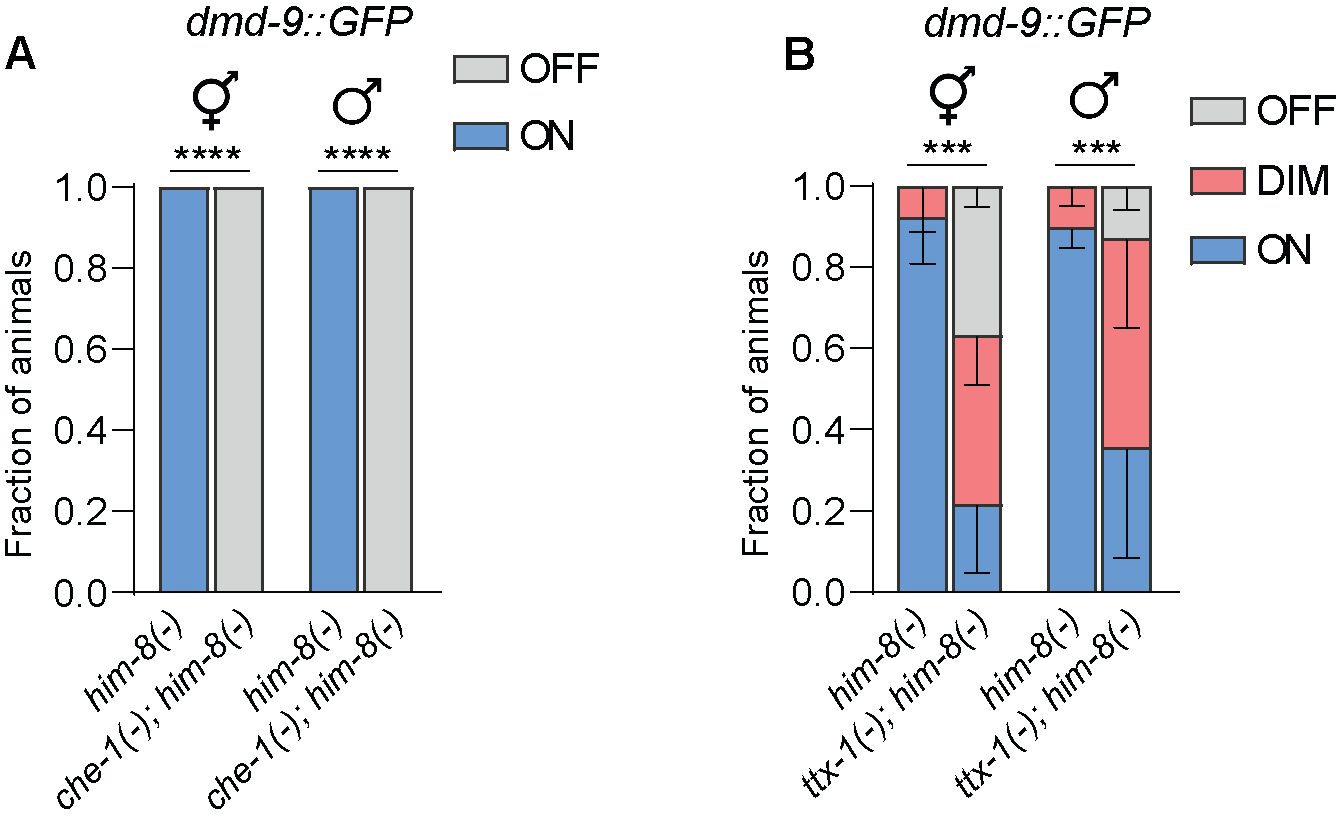

Supplement: jkac305_Supplementary_Data [file jkac305_supplementary_data.zip › Figure_S4_G3-2022-403934.tif]
